# Influence of Conditioning and Expansion Characteristics on the Apparent Metabolizable Energy and Standardized Ileal Amino Acid Digestibility of Full-Fat Soybeans for Broilers

**DOI:** 10.3390/ani12081021

**Published:** 2022-04-14

**Authors:** M. Reza Abdollahi, Markus Wiltafsky-Martin, Faegheh Zaefarian, Velmurugu Ravindran

**Affiliations:** 1 Monogastric Research Center, School of Agriculture and Environment, Massey University, Palmerston North 4442, New Zealand; f.zaefarian@massey.ac.nz (F.Z.); v.ravindran@massey.ac.nz (V.R.); 2 Evonik Operations GmbH, Animal Nutrition, Rodenbacher Chaussee 4, 63457 Hanau, Germany; markus.wiltafsky-martin@evonik.com

**Keywords:** amino acid, broiler, digestibility, full-fat soybean, heat treatment, metabolizable energy

## Abstract

**Simple Summary:**

Full-fat soybean (FFSB) is a valuable source of energy and protein for poultry and is obtained by cracking raw soybeans without cooking or oil extraction. However, due to the presence of trypsin inhibitors and lectins, raw FFSB is not used in poultry diets. These anti-nutritional factors are, however, heat-labile, and a proper hydrothermal process will inactivate these factors, eliminating their adverse effects on nutrient utilization and bird performance. The aim of the present study was to investigate the influence of conditioning and expansion characteristics on the apparent metabolizable energy and standardized ileal digestibility (SID) of amino acids (AA) in FFSB for broilers. Based on the results, long-term conditioning at 100 °C for 6 min followed by expansion with 18 kWh/t specific energy input increased the metabolizable energy and SID AA of FFSB. Further increases in conditioning time above 6 min or expansion of specific energy input above 18 kWh/t did not offer any advantages to metabolizable energy and AA digestibility of FFSB.

**Abstract:**

This study investigated the influence of short-term and long-term conditioning and expansion on the nitrogen-corrected apparent metabolizable energy (AMEn) and standardized ileal digestibility (SID) of amino acids (AA) in full-fat soybeans (FFSB) for broilers. A batch of raw soybeans was used to manufacture 10 FFSB products (T0 to T9) by applying various combinations of conditioning and expansion. The AMEn and SID AA of FFSB were determined by difference and direct methods, respectively. All heat treatments increased (*p* < 0.001) the AMEn compared to raw FFSB. The sample subjected to long-term conditioning at 100 °C for 6 min and expansion at 18 kWh/t (T5) supported 3.88 MJ/kg higher AMEn than the raw FFSB. Raw FFSB had the poorest (*p* < 0.05) AA digestibility. Among the heat-treated samples, the highest (*p* < 0.05) SID AA was recorded for T5. The results demonstrated that the long-term conditioning of FFSB at 100 °C for 6 min prior to expansion with 18 kWh/t specific energy input enhanced metabolizable energy and SID AA. Further increases in conditioning time from 6 to 9 min or expansion of specific energy input from 18 to 28 kWh/t did not yield additional benefits to energy utilization and AA digestibility of FFSB.

## 1. Introduction

Full-fat soybean (FFSB) is obtained by the mechanical cracking of whole soybeans without cooking or oil extraction [1] and can be a good source of energy and protein for poultry [2]. However, the presence of several anti-nutritional factors (ANFs), most importantly trypsin inhibitors (TI) and lectins, is a major obstacle for the inclusion of raw FFSB in poultry diets [3,4,5]. Trypsin inhibitors can bind to proteolytic enzymes, forming inactive complexes and impairing amino acid (AA) digestibility [6]. Most FFSB proteinaceous ANFs are, however, heat-labile, and therefore proper hydrothermal processing, such as conditioning and expansion, will inactivate these factors and eliminate their adverse effects on AA utilization. Multiple research projects have indicated that, once properly processed, broilers can be fed up to 200–250 g FFSB/kg diet without any adverse effect on growth performance [7,8,9,10].

Among the broad spectrum of factors affecting the content and quality of nutrients in FFSB, processing conditions are of the greatest importance. The digestibility of AA in FFSB is influenced to a large extent by the adequacy of heat treatment to inactivate the ANFs. Both under- and over-processing are deleterious to AA digestibility and energy utilization. The feed industry has been monitoring the scope of thermal processing by using in vitro tests such as urease activity, protein solubility in potassium hydroxide (KOH), protein dispersibility index, trypsin inhibitor activity (TIA), and reactive lysine [1]. Raw soybeans usually contain 20–35 mg/g TIA. To eliminate the detrimental effects of TI, TIA needs to be reduced to a level of below 4.0 mg/g [9] or between 1.75 and 2.50 mg/g [1,11]. Excessive heat processing of FFSB has been reported to damage heat-labile AA such as Arg, Cys, and more specifically, Lys through the formation of Maillard reaction products [12,13].

Despite the increasing interest in the use of FFSB in poultry diets, only limited studies have investigated the effects of conditioning and expanding characteristics on the AA digestibility and apparent metabolizable energy (AME) of this feed ingredient [14,15]. It was hypothesized that temperature and time during the conditioning process and different specific energy inputs applied during the expanding process will influence the digestibility of AA and energy utilization by birds. The present study was initiated to investigate the impact of short-term and long-term conditioning and expansion on the nitrogen-corrected AME (AMEn) and standardized ileal digestibility (SID) of AA in FFSB for broiler chickens.

## 2. Materials and Methods

### 2.1. Heat Treatment of Full-Fat Soybeans

A batch of raw soybeans was obtained and used to develop 10 test FFSB samples at the facilities of Rieder Asamhof GmbH & Co. KG (Kissing, Germany). Prior to heat processing, the raw FFSB (T0) were mechanically cracked using a single crumbler (Amandus Kahl GmbH & Co. KG, Typ: D200 × 800). The following equipment was used for the processing of FFSB: short-term (ST) conditioner (Amandus Kahl GmbH & Co. KG, Typ: MK 400 KPL, Reinbek, Germany), long-term (LT) conditioner (Amandus Kahl GmbH & Co. KG, Typ: HR 1201-01, Reinbek, Germany), and expander (Amandus Kahl GmbH & Co. KG, Typ: OE 23.2, Reinbek, Germany). Various combinations of short-term conditioning at 85 °C for 60 s, long-term conditioning at 90 or 100 °C for 3, 6, or 9 min, and expanding with specific energy inputs of 13, 18, and 28 kWh/t were applied to manufacture the 10 FFSB products. All FFSB products were dried in a belt dryer with 105 °C air and they left the dryer after 4 min (Amandus Kahl GmbH & Co. KG, BK 1520-1/22-STOCK, Reinbek, Germany). Then, they entered a belt cooler with ambient air at 25 °C and left it after 6 min.

To produce T1, T2, and T3, ST conditioning was applied to the raw FFSB (T0) for 60 s at a temperature of 85 °C, followed by LT conditioning at 90 °C for 3 min (T1), 6 min (T2), and 9 min (T3), prior to expansion at a specific energy input of 13 kWh/t. The second batch of T0 was conditioned at 85 °C for 60 s (ST conditioning), underwent LT conditioning at 100 °C for 3 min (T4), 6 min (T5), and 9 min (T6), followed by expansion at a specific energy input of 18 kWh/t. The final batch of T0 was ST conditioned, followed by LT conditioning at 100 °C for 3, 6, and 9 min to manufacture T7, T8, and T9, respectively, and then expanded at a specific energy input of 28 kWh/t. The processing conditions for FFSB samples are shown in Table 1.

### 2.2. Assay Diets, Birds, and Housing

The experiments were conducted according to the New Zealand Revised Code of Ethical Conduct for the use of live animals for research, testing, and teaching and approved by the Massey University Animal Ethics Committee.

#### 2.2.1. Determination of Apparent Metabolizable Energy

The AME of FFSB samples was determined by the difference method. In this method, a corn–soybean basal diet was formulated (Table 2) and 10 test diets, each containing different FFSB samples (T0 to T9), were developed by replacing (*w*/*w*) 300 g/kg of the basal diet with one of the FFSB samples. The basal and test diets were offered in mash form.

A total of 396 one-day-old male broiler chicks (Ross 308) were obtained from a commercial hatchery and raised in floor pens until 16 day of age. Birds were fed a commercial broiler starter diet (230 g/kg crude protein and 12.55 MJ/kg AMEn). The temperature was maintained at 31 °C on day 1 and was gradually reduced to 22 °C by the end of the third week. Central ceiling extraction fans and wall inlet ducts controlled the ventilation. The birds received 20 h of fluorescent illumination and were allowed free access to the diets and water. On day 16, the birds were individually weighed, and birds of uniform body weight were randomly allocated to 66 cages (six birds per cage) with six replicates per treatment.

The AME was determined using the total excreta collection procedure from day 16–23 Birds were fed the experimental diets for 7 d (16 to 23 day post-hatch), and the first 3 day served as an adaptation period. The feed intake and total excreta output for each replicate were recorded during the last 4 day of the assay (day 19 to 23). Daily excreta collections were pooled within replicates, mixed well in a blender, and sub-sampled. Sub-samples were lyophilized (Model 0610, Cuddon Engineering, Blenheim, New Zealand), and dried excreta samples were ground to pass through a 0.5 mm sieve and stored in airtight plastic containers at 4 °C pending analysis. The diets and excreta samples were analyzed for dry matter (DM), gross energy (GE), and nitrogen (N).

#### 2.2.2. Determination of Standardized Ileal Digestibility of Amino Acids

A total of 10 assay diets containing different FFSB samples, as the only source of AA in the diet, and dextrose were formulated to contain about 180 g/kg dietary protein (Table 3) [16]. Basal endogenous AA losses (EAA) were measured using a N-free diet (NFD) for the calculation of SID (Table 3). Titanium dioxide (5.0 g/kg; Merck KGaA, Darmstadt, Germany) was added to all diets as an indigestible marker. Each of the 10 assay diets, plus the NFD, was offered in mash form to six cages (six birds per cage) of male Ross 308 broilers. The test diets were offered for 3 days (from day 25 to 28), and birds had ad libitum access to test diets and water.

On day 28, all birds per cage, including those fed the NFD, were euthanized by intravenous injection (0.5 mL per kg body weight) of sodium pentobarbitone solution (Provet NZ Pty. Ltd., Auckland, New Zealand). Digesta were collected from the terminal ileum, as described by Ravindran et al. [17]. The portion of the small intestine from Meckel’s diverticulum to a point about ~40 mm proximal to the ileocecal junction was considered as ileum. The ileal digesta were collected from all birds into a plastic container by gentle flushing with distilled water, pooled within a cage and stored at −20 °C until lyophilized (Model 0610, Cuddon Engineering, Blenheim, New Zealand). The diet and freeze-died digesta samples were ground to pass through a 0.5 mm sieve and stored in air-tight plastic containers at 4 °C until the analysis of DM, titanium (Ti), N, and AA.

### 2.3. Chemical Analysis

The DM was determined using the standard procedure (Method 930.15) [18]. Gross energy was determined by adiabatic bomb calorimeter (Gallenkamp Autobomb, London, UK) standardized with benzoic acid. Nitrogen was analyzed by combustion (Method 968.06) [18] using a CNS-200 carbon, N, sulfur analyzer (LECO Corporation, St. Joseph, MI, USA). The crude protein (CP) content was calculated as N × 6.25. Titanium was measured on an ultraviolet spectrophotometer following the method of Short et al. [19]. The content of reactive lysine (rLys) in the FFSB was determined as outlined by Fontaine et al. [20], based on the transformation of Lys to its AA analog homoarginine by means of guanidination with o-methylisourea. The trypsin inhibitor activity (TIA) was analyzed following method 71–10 (AACC 22–40.01, using DL-BAPA) [21]. The KOH solubility was determined as described by Araba and Dale [22].

Amino acids were analyzed following standard procedures (Method 994.12) [23]. Diet and digesta samples were hydrolyzed in glass tubes in an oven using 6 N HCl (containing phenol) for 24 h at 110 ± 2 °C. An AA analyzer (ion exchange) with ninhydrin post column derivatization was used to measure AA. The chromatograms were integrated using a dedicated software (Agilent Open Lab software, Santa Clara, CA, USA) with AA simultaneously detected at 570 and 440 nm. Cysteine and methionine were determined as cysteic acid and methionine sulphone, respectively, by oxidation with performic acid for 16 h at 0 °C and neutralization with hydrobromic acid prior to hydrolysis. For Trp analysis, the samples were saponified under alkaline conditions with barium hydroxide octahydrate solution in the absence of air at 110 °C for 20 h in an autoclave. Following alkaline hydrolysis, the internal standard α-methyl Trp was added to the mixture. After adjusting the hydrolysate to pH 3.0 and diluting with 30% methanol, Trp and the internal standard were separated by reverse phase chromatography on a HPLC column. Detection was selectively performed with fluorescence detection (extinction 280 nm, emission 356 nm) to prevent interference by other AA and constituents.

### 2.4. Calculations

All data were expressed on a DM basis, and the AME value of FFSB samples were calculated using the following formulas:

AMEdiet (MJ/kg) = [(FI(kg) × GEdiet (MJ/kg)) − (Excreta output(kg) × GEexcreta(MJ/kg))]/FI(kg)(1)

AMEFFSB (MJ/kg) = [AME of FFSB assay diet − (AME of basal diet × 0.70)]/0.30(2)

Nitrogen retention, as a percentage of N intake, was determined as follows:

N retention (%) = 100 × [((FI_(kg)_ × N_Diet(g/kg)_) − (Excreta output_(kg)_ × N_Excreta(g/kg)_))/(FI_(kg)_ × N_Diet(g/kg)_)] (3)

The AME_n_ was then calculated by correction for zero N retention by assuming 36.54 kJ per gram N retained in the body, as described by Titus et al. [24].

N-corrected AME (AME_n_; MJ/kg) = AME − (36.54 × N retention_(g)_)/1000 (4)

The apparent ileal digestibility of AA was calculated from the dietary ratio of AA (%) to Ti (%) relative to the corresponding ratio in the ileal digesta using the following formula.

AID of AA = [[(AA/Ti)_d_ − (AA/Ti)_i_]/(AA/Ti)_d_] × 100 (5)

where (AA/Ti)_d_ = ratio of AA to Ti in the diet; (AA/Ti)_i_ = ratio of AA to Ti in the ileal digesta.

The basal EAA losses from birds fed the NFD were calculated as mg of AA flow per kg of DM intake [25].

Basal EAA flow (mg/kg DM intake) = AA concentration in ileal digesta (mg/kg) × [diet Ti (mg/kg)/ileal digesta Ti (mg/kg)](6)

The calculated basal EAA flow was then used to standardize the apparent AA digestibility data.

SID (%) = AID (%) + [Basal EAA (mg/kg DM intake)/Ing. AA (mg/kg DM)](7)

where SID = standardized ileal digestibility of the AA; AID = apparent ileal digestibility of the AA; Basal EAA = basal endogenous AA loss; and Ing. AA = concentration of the AA in the ingredient.

### 2.5. Data Analysis

The data were analyzed by a one-way ANOVA using the GLM procedure of SAS [26]. The cages were the experimental units. Differences were deemed significant when *p* ≤ 0.05. The Least Significant Difference test was used to compare means.

## 3. Results and Discussion

All birds remained healthy and readily consumed their assay diets throughout the study. No evidence of histopathological abnormalities was observed when the abdominal cavity was opened following euthanasia.

While in vitro protein quality indicators such as urease activity index, protein dispersibility index, KOH protein solubility test, and TIA have been used in the feed industry to evaluate the protein quality of soybean meal, their application to FFSB merits more investigation [2]. Investigating the effect of dry extrusion temperature (115, 125, 135, 145, and 165 °C) on FFSB, Palic et al. [27] concluded that urease activity has a limited application as an indicator of the degree of FFSB processing and should be used to identify only under-processed FFSB. According to Herkelman et al. [28] and Perilla et al. [29], TIA in FFSB is more reflective of its nutritional value and a better predictor of FFSB protein quality than urease activity for chickens. A KOH protein solubility of 78–85% has been suggested for properly heat-treated soybean meals [1,2,22,30]. Moreover, TIA values of below 4.0 mg/g for FFSB [9] and of 1.75–2.50 mg/g for soybean meals are considered necessary to mitigate the negative impact of TI on AA digestibility [1,11]. The in vitro protein quality indicators of the FFSB with different heat treatments are shown in Table 4. Except for the samples LT conditioned at 90 °C (T1, T2, and T3), the KOH protein solubility reduced with LT conditioning at 100 °C for 6 and 9 min, regardless of expanding specific energy input. Expansion with specific energy input of 28 kWh/t resulted in lower KOH protein solubility than those expanded with 18 kWh/t at 3 min (T7 = 92.2 vs. T4 = 96.4), 6 min (T8 = 87.8 vs. T5 = 90.5), and 9 min (T9 = 85.7 vs. T6 = 89.3), with the lowest KOH protein solubility determined in T9. The TIA decreased in all heat-treated samples compared to raw FFSB (T0), with the lowest values recorded for T9 and T6, followed by T5, T8, and T7. The highest TIA recorded for raw FFSB in this study agrees with previous studies demonstrating the negative impact of TI on the digestibility of AA and highlights the fact that raw FFSB must not be used in poultry diets [4,31]. It is also recognized that TI are heat sensitive components and can be effectively reduced or eliminated through the proper heat treatment of raw FFSB. Similar to the current findings, Leeson and Atteh [8] reported a reduction in TIA from 58.7 mg/g in raw soybeans to 16.1, 14.8, 9.3, and 8.4 mg/g following the extrusion of soybeans at 80, 100, 120, and 140 °C, respectively. In the current study, the lowest KOH protein solubility recorded in T9 and the remarkable reduction in TIA in T9 and T6 might suggest that LT conditioning at 100 °C for 9 min is required to properly deactivate the TI and is more efficient when combined with a specific energy input of 28 kWh/t during the expansion. The T9 sample with 3.9 mg/g TIA and 85.7% KOH protein solubility was the only heat-treated sample in the current study to meet the suggested maximum for TIA (4.0 mg/g) [9] and KOH protein solubility (78–85%) [2] in adequately heat-treated soybean products.

The AA composition of raw FFSB used in the present study was within the range previously reported [2,15,32]. The ST conditioning and LT conditioning at different temperatures and times, and expansion with different specific energy inputs, had no notable effects on the contents of Lys and rLys (Table 4), CP, and individual and total AA (Table 5) in FFSB samples. This finding suggests that the heat treatments applied had minimal impact on the gross content of AA and mainly influence the nutritional quality through their impact on digestibility of nutrients [33]. Severe thermo-mechanical treatments can favor the formation of Maillard reaction products [20,34,35]. Free amino groups from AA, the epsilon-amino group, and free aldehyde groups from reducing sugars can interact and result in the destruction of some AA, Lys in particular, that can be characterized by the reduction in rLys [20]. The lack of ST and LT conditioning effect on the concentration of Lys, rLys, and rLys:Lys ratio in this study suggests that the heat processing conditions applied in this study were not destructive to any AA.

Despite increasing interest in the use of FFSB in poultry diets, there are only sporadic data on the influence of heat processing conditions on the AMEn and SID AA of FFSB for poultry [14,15]. The influence of heat treatment on the AME, AMEn, N retention, and SID AA of FFSB in broilers is summarized in Table 6. All heat treatment processes investigated in this study significantly (*p* < 0.001) increased the AME, AMEn, and N retention in FFSB samples compared to raw FFSB (T0). The raw FFSB showed the lowest (*p* < 0.05) AME, AMEn, and N retention. Among the heat-treated samples, T3, T5, T7, T8, and T9 showed similar (*p* > 0.05) AME and AMEn values but were higher (*p* < 0.05) than other samples. The highest (*p* < 0.05) N retention was recorded in T5, followed by T3 and T8. Among all heat-treated samples, the smallest improvements in AME, AMEn, and N retention were achieved in T2 and T4. Compared to the raw sample, T5 supported 4.49 and 3.88 MJ/kg higher AME (15.54 versus 11.05 MJ/kg) and AMEn (14.29 versus 10.41 MJ/kg), respectively. No further improvements in AME or AMEn were observed with increases in LT conditioning time and/or expansion of specific energy input above T5 (LT conditioned at 100 °C for 6 min and expanded with 18 kWh/t specific energy input). The high concentration of fat and its digestion extent make major contributions to metabolizable energy of FFSB. As indicated by Kan et al. [14], the increase in AMEn because of heat treatment could be attributed to an increase in digestibility of fat, and perhaps protein, in FFSB.

A significant (*p* < 0.001) effect of heat treatment on the SID of protein, all individual indispensable (IAA), dispensable (DAA), and average of all AA was observed (Table 6). The raw FFSB (T0) had the poorest (*p* < 0.05) digestibility of protein and all individual AA, highlighting the documented fact that raw FFSB should be avoided in poultry diets [4,31]. Among the heat-treated samples, the lowest SID protein and average of all AA was recorded for T2, followed by T4, T1, and T3. When heat-treated, the highest (*p* < 0.05) SID for all indispensable AA was recorded for T5, the lowest (*p* < 0.05) SID for T2 and T4, with the other heat-treated samples being intermediate. A similar pattern was observed for all dispensable AA, with T5 and T2 generating the greatest (*p* < 0.05) and poorest (*p* < 0.05) digestibility among heat-treated samples, respectively, with other samples being intermediate.

Similar to patterns observed for the AME, AMEn, and N retention, the highest SIDs of CP and AA were recorded in T5 and confirm that, under the conditions of the present study, the LT conditioning at 100 °C for 6 min prior to expansion with 18 kWh/t specific energy input is sufficient to improve the SID of CP and AA in FFSB. It is pertinent to note that despite a striking increase of 45% in average SID of AA in T5 (78.3%) compared to T0 (54.0%), the KOH protein solubility only declined by 2.8 percentage points (93.3 vs. 90.5%) and to a level beyond the industry recommended KOH protein solubility of 78–85% for properly heat-treated soybean meals [2,22]. These findings suggest that (i) guidelines for KOH protein solubility suggested for soybean meal might not be applicable to FFSB, and (ii) KOH protein solubility is not a good indicator to assess whether the FFSB has been properly processed, and therefore should not be used as the sole measure of optimal processing and AA digestibility in FFSB. Interestingly, increases in LT conditioning time from 6 to 9 min or expansion of specific energy input from 18 to 28 kWh/t, although reducing the KOH protein solubility and TIA further, did not lead to any extra benefits to AA digestibility. It is also noteworthy that the highest average SID of all AA and individual AA observed in T5 did not correspond to the lowest values for KOH protein solubility and TIA, suggesting that broiler chickens might be tolerant to higher levels of TI (TIA of 10.6 mg/g in T5) in FFSB than the previously reported optimum value of <4.0 mg/g [9]. Further deactivation of TI in T9 (TIA of 3.90 mg/g) failed to benefit AA digestibility. In contrast, Clarke and Wiseman [9] measured the digestibility of AA in FFSB samples with different TIA contents of 14.8, 9.6, 4.5, and 1.9 mg/g, and recorded the highest AA digestibility in the FFSB sample with TIA of 1.9 mg/g.

Despite numerical reductions in the SID AA, intensifying the level of heat treatment beyond T5 (LT conditioned at 100 °C for 6 min and expanded with 18 kWh/t energy input) did not significantly (*p* > 0.05) deteriorate the AA digestibility. However, it should be recognized that heat-induced structural changes in an AA might not be accurately captured by the digestibility measurements as the damaged AA can be digested and absorbed without the ability to participate in metabolic reactions in the animal body [36]. Nevertheless, Oliveira et al. [37] reported no effect of different FFSB extrusion temperatures (125, 130, 135, and 140 °C) on the performance and carcass composition of broilers (22–45 d) and suggested that FFSB extruded at temperatures of 125 to 140 °C can be used in broiler diets after 22 d of age. Similarly, in the study by Herkelman et al. [28], maximum broiler performance was achieved when FFSB was heated at 121 °C for 40 min compared to heating times of 10, 20, 30, 60, and 90 min. Perilla et al. [29] concluded that the optimum heating temperature during the wet extrusion of FFSB for broilers lies between 122 °C and 126 °C.

## 4. Conclusions

The findings of the current study demonstrated that long-term conditioning of FFSB at 100 °C for 6 min prior to expansion with 18 kWh/t specific energy input enhanced the AMEn and SID of protein and AA. Further increases in long-term conditioning time from 6 to 9 min or expansion of specific energy inputs from 18 to 28 kWh/t, while reducing KOH protein solubility and TIA, did not deliver additional benefits to energy utilization and AA digestibility.

## Figures and Tables

**Table 1:** Characteristics of heat treatment of full-fat soybeans.

Conditioning							
Sample	Cracked	Short-Term	Long-Term Temperature		Specific Energy Input		
		85 °C, 60 s	90 °C	100 °C	13 kWh/t	18 kWh/t	28 kWh/t
T0	+	-	-	-	-	-	-
T1	+	+	3 min	-	+	-	-
T2	+	+	6 min	-	+	-	-
T3	+	+	9 min	-	+	-	-
T4	+	+	-	3 min	-	+	-
T5	+	+	-	6 min	-	+	-
T6	+	+	-	9 min	-	+	-
T7	+	+	-	3 min	-	-	+
T8	+	+	-	6 min	-	-	+
T9	+	+	-	9 min	-	-	+

kWh/t, kilowatt hour/t. Total of 10 samples were used in the experiment: T0, T1, T2, T3, T4, T5, T6, T7, T8, and T9. The temperatures achieved at the end of the expander with the different energy inputs of 13, 18, and 28 kWh/t were 90 °C, 110 °C, and 120 °C, respectively. The materials needed about 15 s to pass through the expander. After heat treatment, all full-fat soybeans were dried in a belt dryer with 105 °C air and left the dryer after 4 min. Then, they entered the belt cooler using ambient air of 25 °C and left it after 6 min.

**Table 2:** Composition (g/kg, as fed basis) of the basal diet used in the metabolizable energy assay.

Item	Inclusion (g/kg)
Corn	612.4
Soybean meal	351.8
Dicalcium phosphate	21.7
Limestone	7.8
Sodium chloride	2.0
Sodium bicarbonate	2.3
Vitamin and trace mineral premix ^1^	2.0

^1^ Supplied per kilogram of diet: antioxidant, 100 mg; biotin, 0.2 mg; calcium pantothenate, 12.8 mg; cholecalciferol, 60 µg; cyanocobalamin, 0.017 mg; folic acid, 5.2 mg; menadione, 4 mg; niacin, 35 mg; pyridoxine, 10 mg; trans-retinol, 3.33 mg; riboflavin, 12 mg; thiamine, 3.0 mg; dl-α-tocopheryl acetate, 60 mg; choline chloride, 638 mg; Co, 0.3 mg; Cu, 3.0 mg; Fe, 25 mg; I, 1 mg; Mn, 125 mg; Mo, 0.5 mg; Se, 200 µg; Zn, 60 mg.

**Table 3:** Composition of the test diet and nitrogen-free diet (NFD) (g/kg, as fed basis) used in the amino acid digestibility assay.

Item	Inclusion (g/kg)	
	Test Diet	NFD
Full-fat soybean	500	-
Dextrose	440	869
Soybean oil	20.0	50.0
Dicalcium phosphate	19.0	19.0
Limestone	10.0	13.0
Titanium dioxide ^1^	5.0	5.0
Sodium chloride	2.0	3.0
Sodium bicarbonate	2.0	3.0
Vitamin and trace mineral premix ^2^	2.0	2.0
Cellulose ^3^	-	35.0
Dipotassium phosphate	-	1.0

^1^ Merck KGaA, Darmstadt, Germany. ^2^ Supplied per kilogram of diet: antioxidant, 100 mg; biotin, 0.2 mg; calcium pantothenate, 12.8 mg; cholecalciferol, 60 µg; cyanocobalamin, 0.017 mg; folic acid, 5.2 mg; menadione, 4 mg; niacin, 35 mg; pyridoxine, 10 mg; trans-retinol, 3.33 mg; riboflavin, 12 mg; thiamine, 3.0 mg; dl-α-tocopheryl acetate, 60 mg; choline chloride, 638 mg; Co, 0.3 mg; Cu, 3.0 mg; Fe, 25 mg; I, 1 mg; Mn, 125 mg; Mo, 0.5 mg; Se, 200 µg; Zn, 60 mg. ^3^ Ceolus™, Microcrystalline Cellulose, Asahi Kasei Corporation, Tokyo, Japan.

**Table 4:** Effect of heat treatments on the in vitro protein quality indicators of full-fat soybeans.

Item	T0	T1	T2	T3	T4	T5	T6	T7	T8	T9
DM	917	900	898	898	914	911	910	921	921	922
KOH (%)	93.3	87.4	90.7	91.9	96.4	90.5	89.3	92.2	87.8	85.7
TIA (mg/g)	32.7	15.2	21.6	16.0	24.3	10.6	7.6	12.1	11.0	3.90
Lys (g/kg, as received basis)	23.8	23.2	23.6	24.0	23.2	23.0	23.2	23.4	23.2	23.3
rLys (g/kg, as received basis)	21.6	21.3	21.5	21.8	21.4	20.9	21.1	21.6	21.3	21.4
rLys:Lys (%)	90.8	91.7	91.3	91.1	92.1	90.9	91.0	92.6	91.7	91.8

See Table 1 for details of heat treatments. KOH = protein solubility in 0.2% potassium hydroxide; TIA = trypsin inhibitor activity; DM = dry matter. Lys = lysine; rLys = reactive lysine.

**Table 5:** Analyzed amino acid (AA) contents (g/kg, dry matter basis) of differently heat-treated full-fat soybeans ^1^.

Item	T0	T1	T2	T3	T4	T5	T6	T7	T8	T9
Crude protein	404	413	416	417	411	411	410	413	411	408
Indispensable AA										
Arg	29.4	29.7	30.3	30.7	30.1	30.3	30.0	30.4	30.0	30.0
His	10.4	10.6	10.6	10.7	10.6	10.6	10.6	10.7	10.5	10.5
Ile	18.2	18.5	18.5	18.9	18.4	18.7	18.4	18.3	17.9	18.1
Leu	29.9	30.5	30.6	30.9	30.5	30.8	30.5	30.8	30.4	30.4
Lys	25.5	25.7	25.8	26.0	25.8	25.9	25.7	25.9	25.7	25.5
Met	5.42	5.55	5.22	5.54	5.65	5.58	5.43	5.63	5.49	5.45
Phe	19.8	20.2	20.2	20.5	20.0	20.4	20.1	20.3	20.0	20.1
Thr	15.6	15.8	15.7	15.7	15.8	15.8	15.8	16.0	16.0	15.8
Trp	5.21	5.41	5.38	5.50	5.33	5.29	5.36	5.27	5.33	5.39
Val	19.0	19.2	19.3	19.7	19.2	19.4	19.1	19.0	18.5	18.9
Dispensable AA										
Ala	17.3	17.4	17.5	17.6	17.5	17.7	17.5	17.7	17.6	17.5
Asp	45.2	46.3	46.1	46.8	46.1	46.5	46.1	46.9	46.3	46.0
Cys ^2^	6.47	6.42	6.38	6.33	6.44	6.31	6.37	6.54	6.33	6.31
Glu	70.4	72.6	72.5	73.2	72.4	73.0	72.4	73.4	72.5	72.2
Gly ^2^	16.9	17.0	17.1	17.2	17.1	17.1	17.0	17.3	17.0	17.0
Pro	20.3	20.5	20.5	20.7	20.0	20.4	20.9	20.7	20.4	20.3
Ser	20.0	20.4	20.4	20.1	20.2	20.3	20.4	20.9	21.0	20.6
Total AA	375	382	382	386	381	384	382	386	381	380

^1^ See Table 1 for details of heat treatments. ^2^ Semi-indispensable amino acids for poultry. Arg = arginine; His = histidine; Ile = isoleucine; Leu = leucine; Lys = lysine; Met = methionine; Phe = phenylalanine; Thr = threonine; Trp = tryptophan; Val = valine; Ala = alanine; Asp = aspartic acid; Cys = cysteine; Glu = glutamic acid; Gly = glycine; Pro = proline; Ser = serine.

**Table 6:** The influence of heat treatments on the apparent metabolizable energy (AME), nitrogen-corrected AME (AMEn), nitrogen retention, and standardized ileal digestibility ^1^ (SID; %) of protein and amino acids (AA; %) of full-fat soybeans in broilers ^2,3^.

Item	T0	T1	T2	T3	T4	T5	T6	T7	T8	T9	Pooled SEM	*p*-Value
AME (MJ/kg DM)	11.05 c	14.74 ab	13.79 b	15.31 a	13.94 b	15.54 a	14.86 ab	15.18 a	15.15 a	15.15 a	0.379	0.001
AME_n_ (MJ/kg DM)	10.41 c	13.62 ab	12.79 b	14.10 a	12.97 b	14.29 a	13.75 ab	14.04 a	13.94 a	14.09 a	0.341	0.001
Nitrogen retention (% of intake)	40.6 e	49.2 abc	47.1 cd	50.5 ab	45.5 d	51.7 a	49.1 abc	49.6 abc	50.4 ab	48.2 bcd	1.142	0.001
SID of protein	55.5 d	72.8 ab	66.0 c	73.1 ab	69.4 bc	77.5 a	74.3 ab	75.0 a	76.3 a	74.6 ab	1.91	0.001
SID of indispensable AA												
Arg	66.8 c	81.0 a	74.8 b	80.8 a	79.5 a	83.8 a	80.5 a	81.0 a	82.5 a	80.9 a	1.64	0.001
His	64.2 c	78.1 ab	72.5 b	79.8 a	77.7 ab	82.9 a	80.9 a	77.7 ab	81.8 a	81.3 a	1.98	0.001
Ile	47.5 e	71.1 bc	59.6 d	70.5 bc	65.6 cd	77.4 a	75.0 ab	74.8 ab	75.8 ab	75.0 ab	2.13	0.001
Leu	50.3 e	71.3 bc	61.2 d	71.3 bc	67.0 cd	77.3 a	74.8 ab	74.8 ab	76.1 ab	74.9 ab	2.10	0.001
Lys	64.9 c	78.8 a	72.0 b	79.2 a	78.0 a	82.5 a	80.1 a	80.7 a	81.6 a	80.5 a	1.71	0.001
Met	54.3 d	77.3 ab	68.3 c	78.6 a	72.7 bc	82.1 a	79.6 a	78.8 a	79.9 a	78.9 a	1.81	0.001
Phe	56.3 e	76.8 abc	66.7 d	76.2 bc	72.6 c	81.9 a	77.5 abc	77.3 abc	78.3 ab	76.9 abc	2.00	0.001
Thr	48.4 e	66.6 bc	60.0 d	68.8 abc	64.9 cd	73.1 a	70.4 abc	70.9 ab	72.2 ab	70.4 abc	2.06	0.001
Trp	45.2 c	70.2 a	59.2 b	69.7 a	60.9 b	75.2 a	75.0 a	73.4 a	74.5 a	73.8 a	2.02	0.001
Val	49.3 e	71.4 ab	60.2 d	70.3 bc	65.2 cd	76.8 a	74.6 ab	74.3 ab	75.1 ab	74.4 ab	2.09	0.001
SID of dispensable AA												
Ala	53.3 a	71.9 ab	63.2 c	72.1 ab	68.3 bc	77.5 a	74.9 a	75.0 a	76.0 a	75.0 a	1.99	0.001
Asp	56.2 d	73.0 ab	66.6 c	74.2 ab	71.0 bc	78.4 a	75.7 ab	76.5 a	77.5 a	75.6 ab	1.90	0.001
Cys ^4^	39.5 f	62.8 cd	53.9 e	64.5 bcd	58.0 de	71.0 a	69.4 ab	68.1 abc	70.4 ab	68.9 abc	2.30	0.001
Glu	64.6 c	78.8 a	72.6 b	79.1 a	77.6 a	82.0 a	79.0 a	79.4 a	80.5 a	79.1 a	1.71	0.001
Gly ^4^	50.4 e	69.3 bc	61.5 d	69.9 abc	65.9 cd	75.1 a	72.3 ab	72.3 ab	73.3 ab	72.2 ab	2.02	0.001
Pro	58.0 d	73.8 ab	67.7 c	74.9 ab	70.4 bc	77.6 a	75.4 ab	75.3 ab	77.3 a	74.7 ab	1.90	0.001
Ser	48.1 e	68.8 bc	62.2 d	71.6 abc	66.7 cd	75.8 a	73.5 ab	74.0 ab	75.9 a	73.6 ab	2.06	0.001
Average SID of all AA	54.0 d	73.0 ab	64.8 c	73.6 ab	69.5 bc	78.3 a	75.8 a	75.5 a	77.0 a	75.6 a	1.91	0.001

Means in a row not sharing a common letter (a–f) are different (*p* < 0.05). Arg = arginine; His = histidine; Ile = isoleucine; Leu = leucine; Lys = lysine; Met = methionine; Phe = phenylalanine; Thr = threonine; Trp = tryptophan; Val = valine; Ala = alanine; Asp = aspartic acid; Cys = cysteine; Glu = glutamic acid; Gly = glycine; Pro = proline; Ser = serine. ^1^ Apparent digestibility values were standardized using the following basal ileal endogenous flow values (g/kg DM intake), determined following the feeding of a nitrogen-free diet: crude protein, 6.1; Arg, 0.17; His, 0.06; Ile, 0.18; Leu, 0.27; Lys, 0.12; Met, 0.05; Phe, 0.15; Thr, 0.39; Trp, 0.06; Val, 0.25; Ala, 0.18; Asp, 0.39; Cys, 0.13; Glu, 0.45; Gly, 0.22; Pro, 0.29; and Ser, 0.30. ^2^ Each value represents the mean of six replicates (six birds per replicate). ^3^ See Table 1 for details of heat treatments. ^4^ Semi-indispensable amino acids for poultry.

## Data Availability

All available data are incorporated into the manuscript.
